# Supplementary material for: The functional small RNA interactome reveals targets for the vancomycin-responsive sRNA RsaOI in vancomycin-tolerant Staphylococcus aureus
Source: mSystems. 2024 Mar 27;9(4):e00971-23. doi: 10.1128/msystems.00971-23 (PMC11019875; doi:10.1128/msystems.00971-23)
Supplement: Supplemental Figures — Figures S1 to S7. [file msystems.00971-23-s0001.pdf]

Supplementary Figure 1

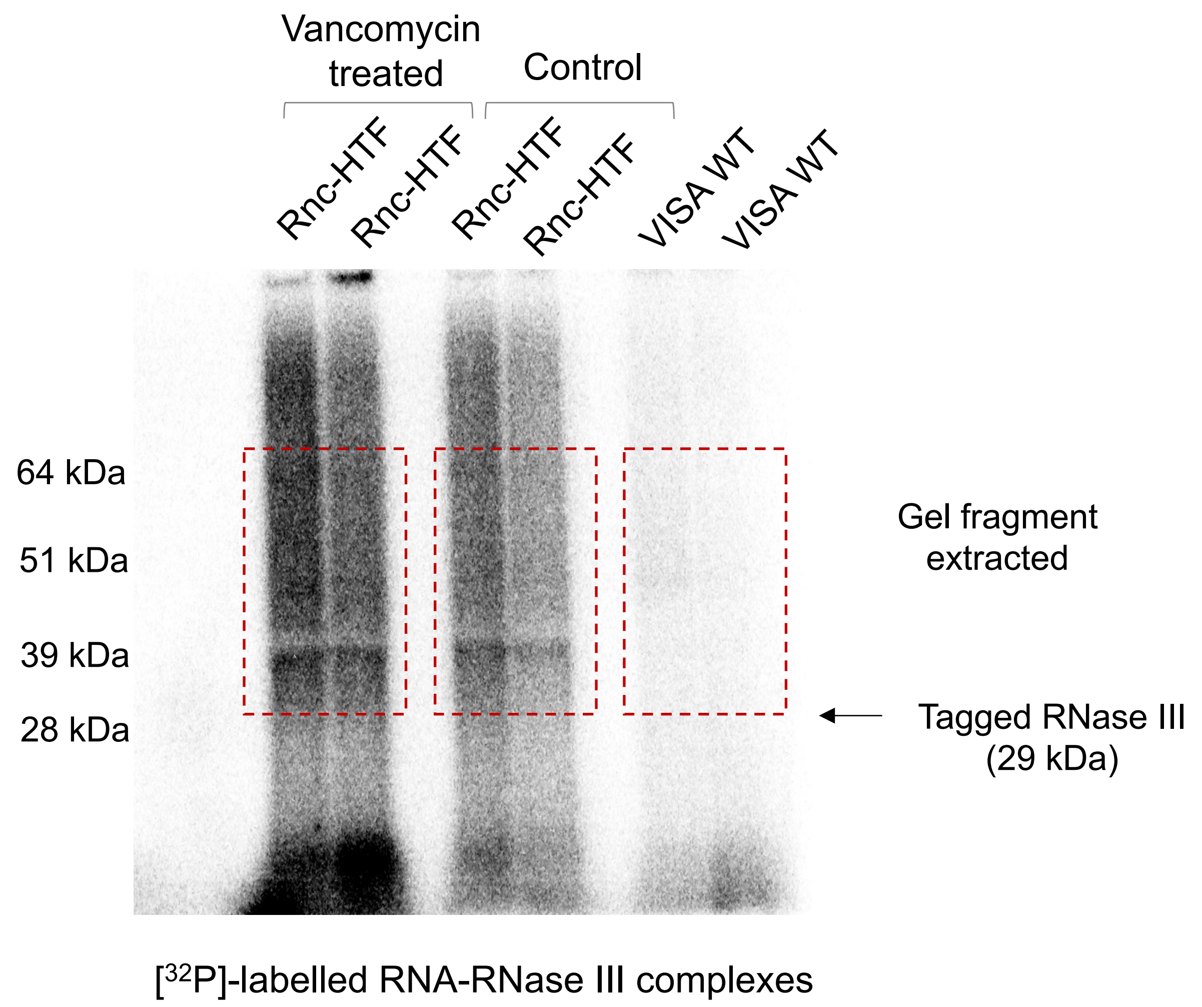

Supplementary Figure 1. Autoradiogram of recovered RNA-RNase III complexes after affinity purification. Autoradiogram signal was observed after 2 hours of exposure and gel fragments from 28 kDa to 64 kDa outlined in the red box were extracted. Lane 1: vancomycin treated Rnc-HTF JKD6008 replicate 1. Lane 2: vancomycin treated Rnc-HTF JKD6008 replicate 2. Lane 3: untreated Rnc-HTF JKD6008 replicate 1. Lane 4: untreated Rnc-HTF JKD6008 replicate 2. Lane 5: wildtype JKD6008 replicate 1. Lane 6: wildtype JKD6008 replicate 2.

Supplementary Figure 2

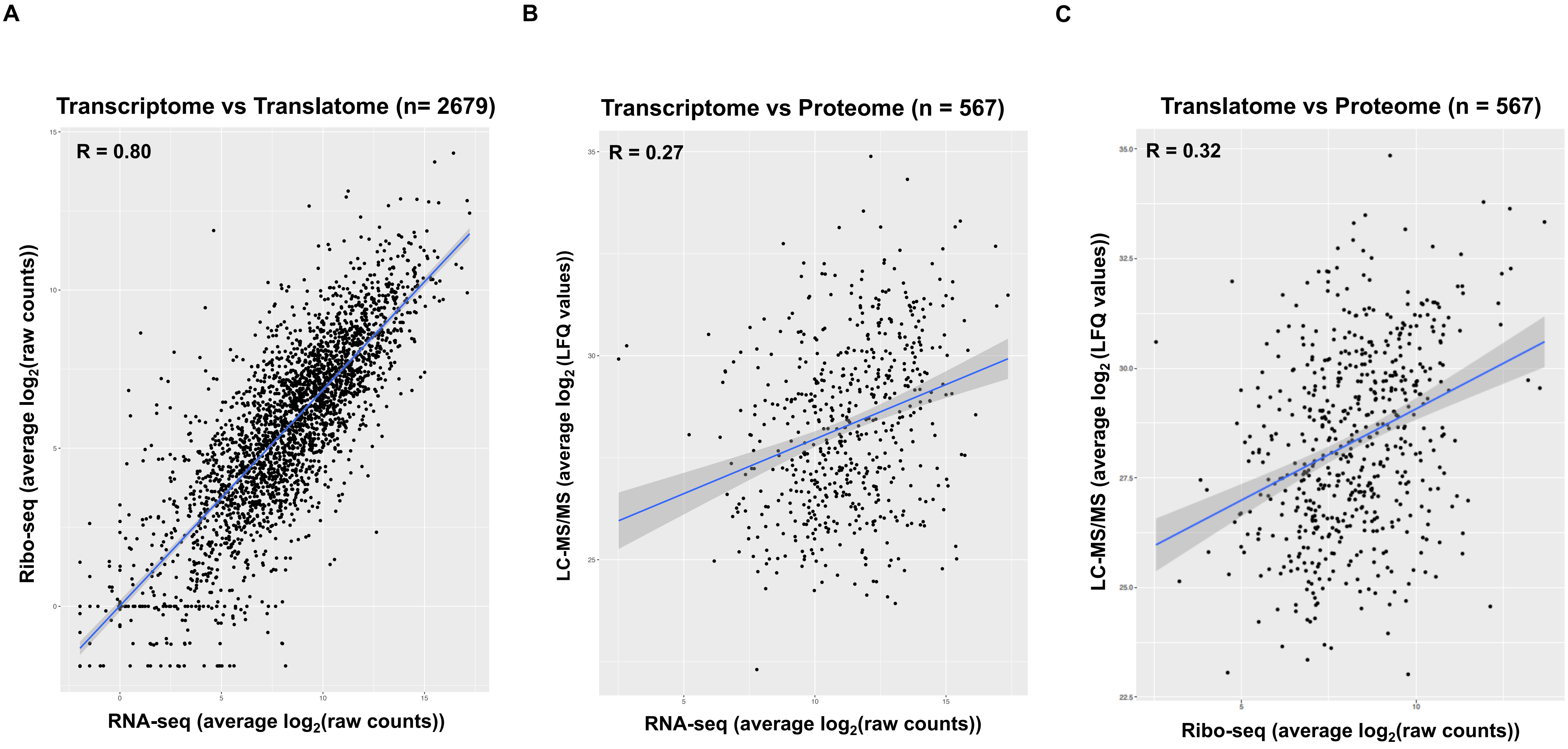

Supplementary Figure 2. Correlations of gene expression levels between the RNAseq (transcriptome), Ribo-seq (translatome), and mass spectrometry (proteome) samples. (A) Average transcript abundance versus average ribosome occupancy. **(B)** Average transcript abundance versus average protein levels. **(C)** Average ribosome occupancy versus average protein levels.

Supplementary Figure 3

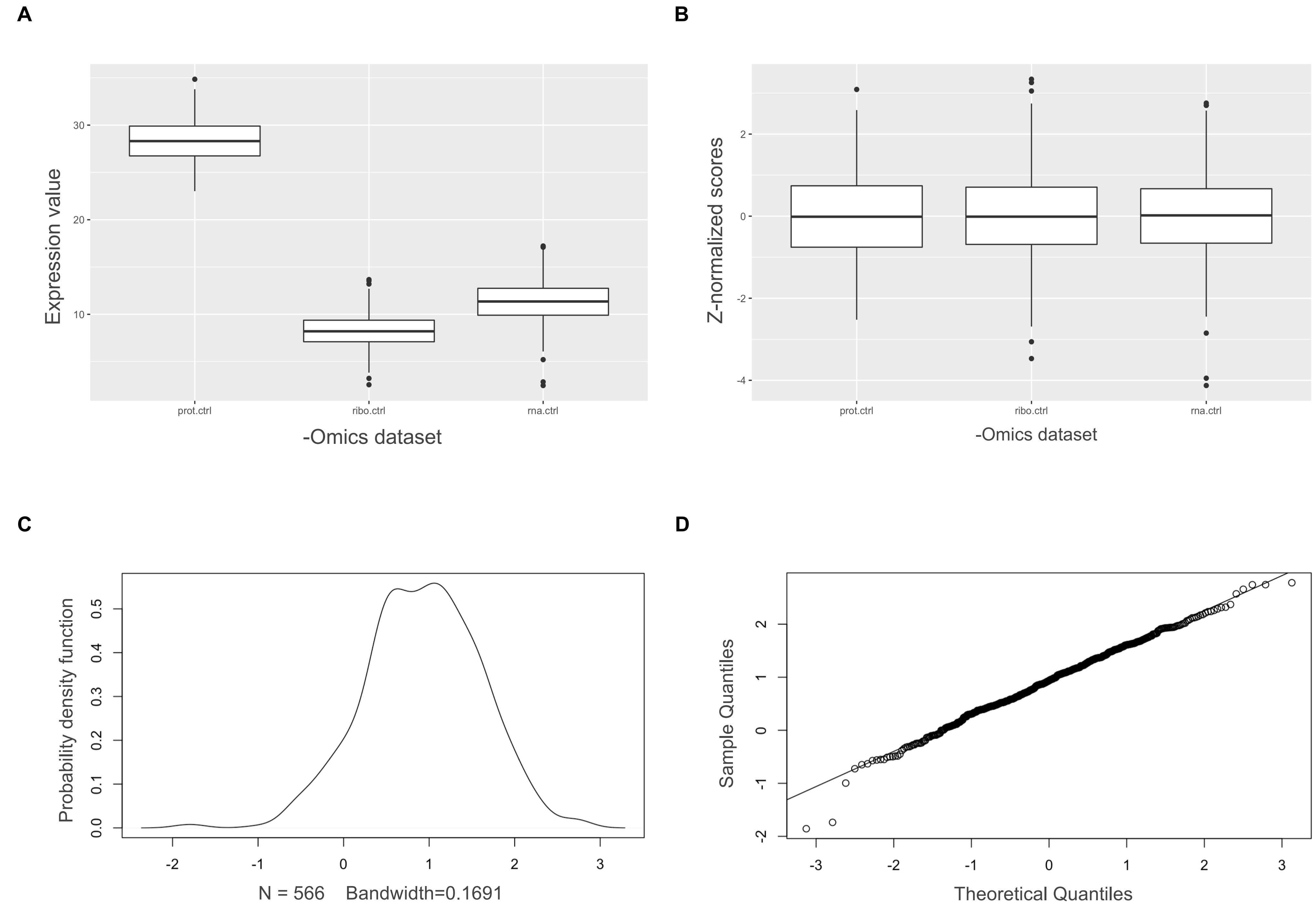

Supplementary Figure 3. Quality control of the multi-omics datasets prior to SOMs clustering. (A) Boxplots of the average gene expression values for each -omics dataset prior to z-score normalisation. The expression values for the RNA-seq and Ribo-seq are represented as $\log_2$(raw counts) while the proteomics are represented as $\log_2$(label-free quantification value). (B) Boxplots of z-normalised values for each -omics condition. (C) Kernel-density plot and normal Q-Q plot (D) for the multi-omics datasets post z-normalisation.

Supplementary Figure 4

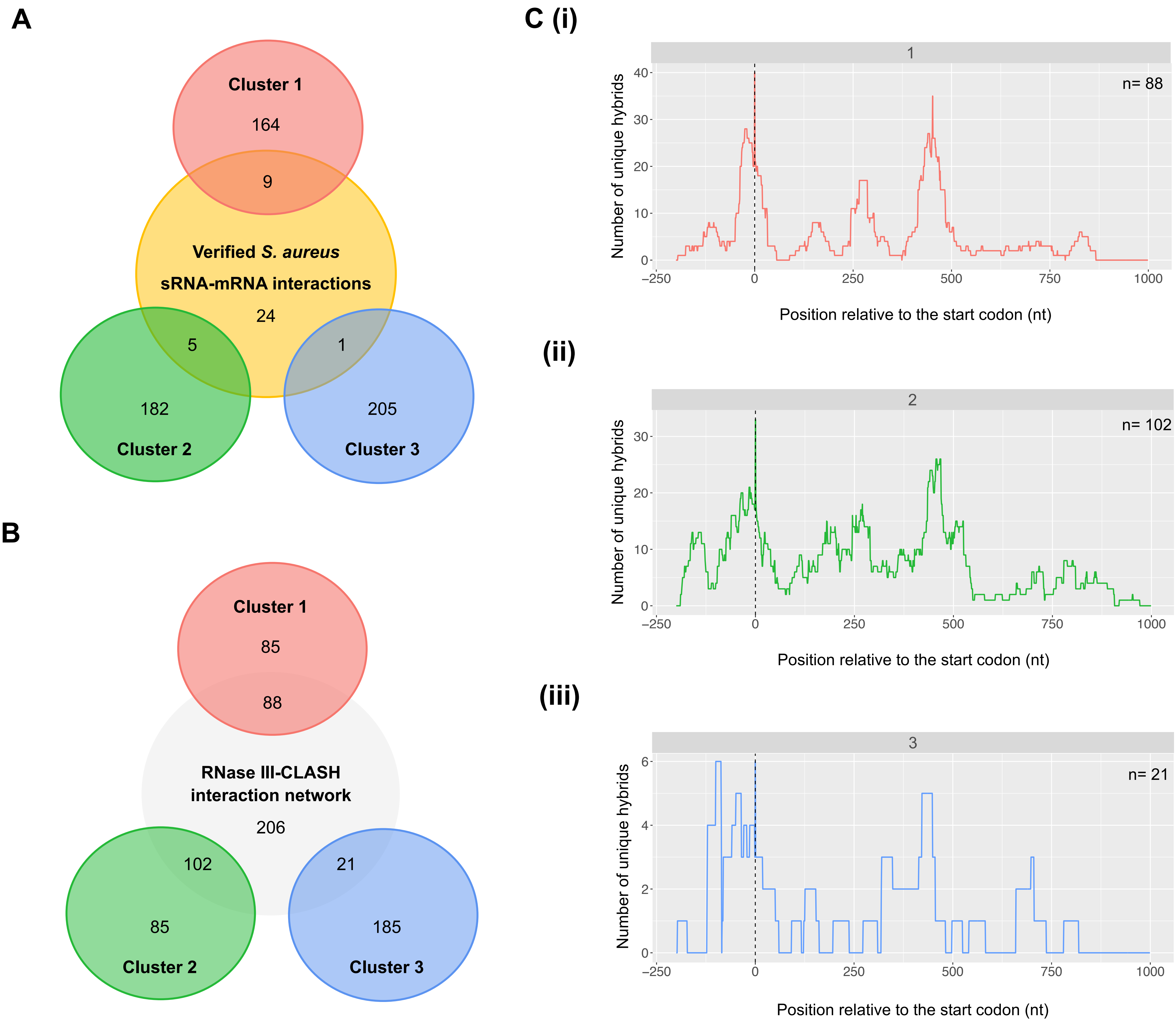

Supplementary Figure 4. ***S. aureus*** **RNase III-CLASH captured sRNA-mRNAi interactions overlaid with 1x3 SOMs clusters (A)** Venn diagram of the 39 verified *S. aureus* sRNA-mRNA interactions overlayed with the SOMs clusters based on the mRNA targets. **(B)** Venn diagram of the 465 RNase-III CLASH captured sRNA-mRNA interactions overlayed with the SOMs clusters based on the mRNA targets. **(C)** A metagene plot was generated for sRNA-mRNA interactions that were detected in clusters 1 **(i)**, 2 **(ii)**, and 3 **(iii)**.

Supplementary Figure 5

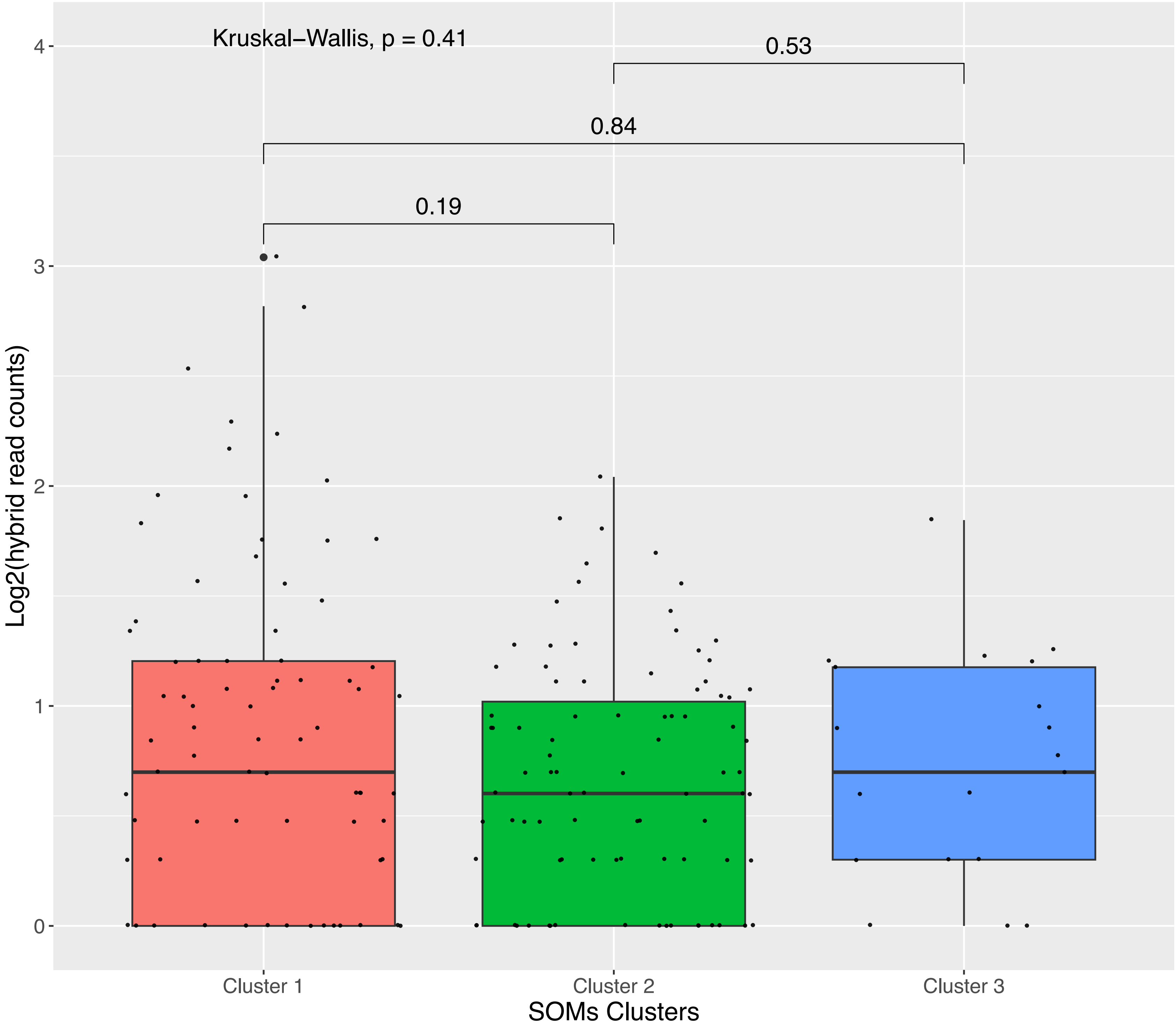

Supplementary Figure 5. Distribution of sRNA-mRNA total hybrid counts in the 1x3 SOMs clusters. A boxplot was generated for sRNA-mRNA total hybrid counts that were detected in cluster 1, 2, and 3 (x-axis). The y-axis represents hybrid read count (log2).

Supplementary Figure 6

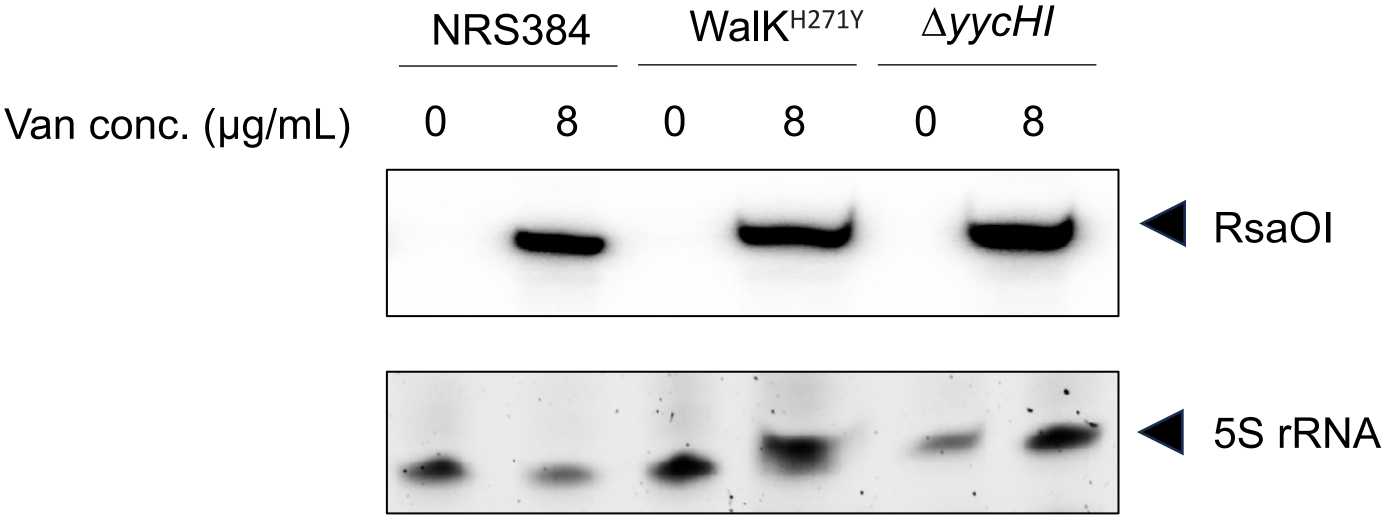

Supplementary Figure 6. Northern blot analysis of RsaOI abundance in NRS384, WalK^{H271Y}, and Δ*yyCHI*. Total RNA was extracted from the indicated strains treated with or without 8 µg/mL vancomycin for 30 minutes and then probed for RsaOI, All strains are derived from the USA300 strain NRS384.

Supplementary Figure 7

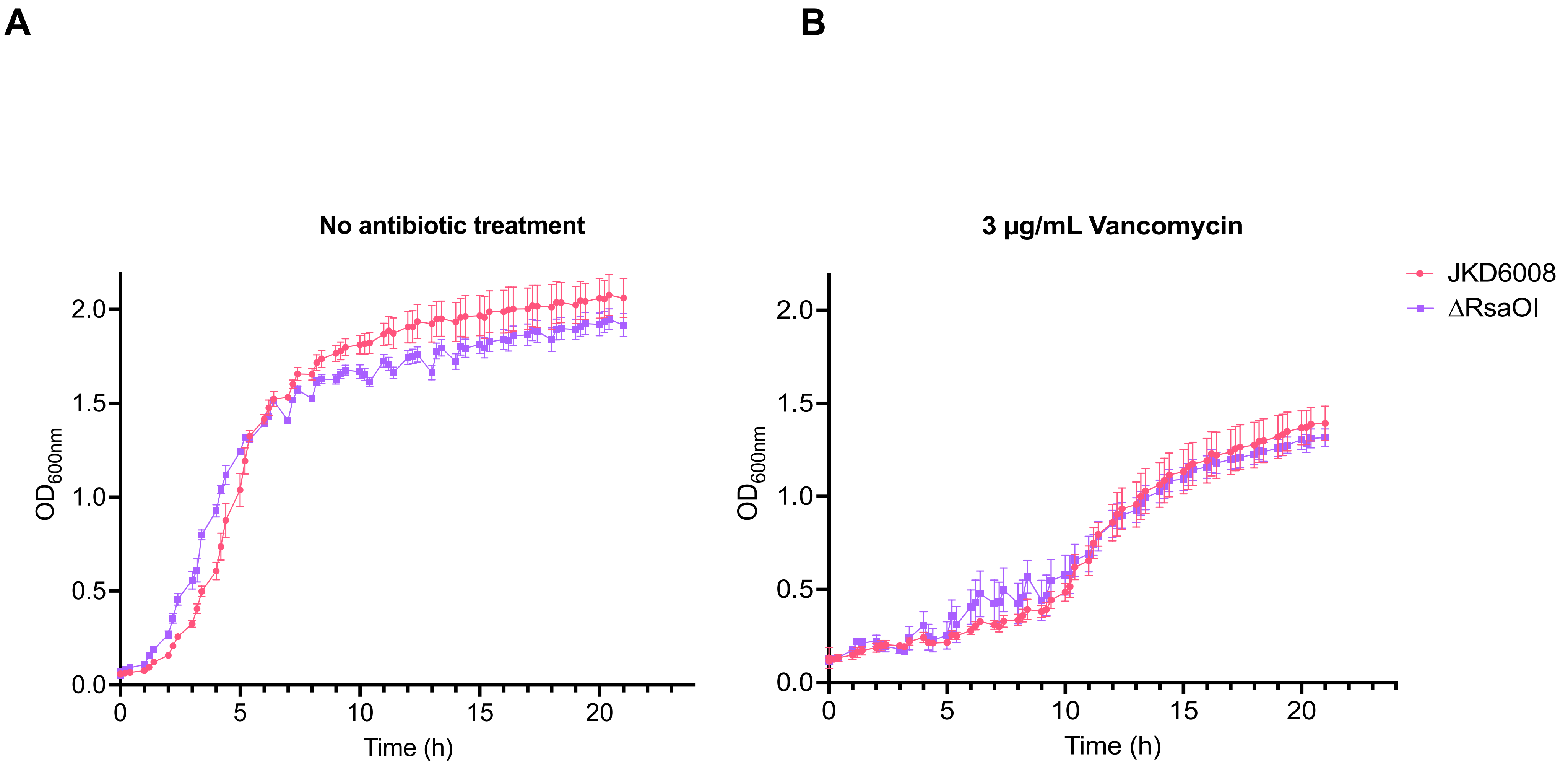

Supplementary Figure 7. Growth curve analysis of VISA (JKD6008) and this isogenic Δ*rsaOI* mutant. Cultures were grown in MH media supplemented with or without 3 µg/mL vancomycin.
